# Functional and structural plasticity induced by audiovisual associations and sensory experiences

**DOI:** 10.1007/s00429-025-02951-3

**Published:** 2025-06-08

**Authors:** Fazilet Zeynep Yildirim-Keles, Pinar Demirayak, Hulusi Kafaligonul

**Affiliations:** 1 Department of Psychology, Boğaziçi University, Istanbul, Türkiye; 2 Department of Psychology, University of Fribourg, Fribourg, Switzerland; 3 Civitan International Research Center, University of Alabama at Birmingham, Birmingham, AL 35233 USA; 4 Department of Neurobiology, University of Alabama at Birmingham, Birmingham, AL 35233 USA; 5 Neuroscience and Neurotechnology Center of Excellence (NÖROM), Department of Anatomy, Faculty of Medicine, Gazi University, Ankara, Türkiye; 6 Department of Neuroscience, Aysel Sabuncu Brain Research Center, Bilkent University, Ankara, Türkiye

**Keywords:** Crossmodal associations, Motion perception, Sensory experiences, Resting-state, Functional connectivity, Cortical thickness, Neural plasticity

## Abstract

Crossmodal associations and correspondences play important roles in shaping perception and guiding our actions. However, we still have a limited understanding of association-induced changes in the adult human brain. An important question to address is to what extent passive exposure to crossmodal associations leads to functional and structural changes in the brain. Utilizing an audiovisual association paradigm in which motion stimuli presented in the left visual field were paired with auditory tones, we investigated experience-dependent crossmodal plasticity by examining resting-state functional connectivity and cortical thickness. Following the association phase, we observed widespread increases in functional connectivity between the trained region (e.g., right V1, V2, and V3) and higher-order frontal, temporal, and occipital areas. In contrast, functional connectivity changes in the untrained region (e.g., left V1, V2, and V3) were markedly reduced, indicating that the observed plasticity was largely specific to the cortical representation of the stimulated visual field. Consistent with the functional results, cortical thickness analysis revealed a clear hemispheric asymmetry, with significant changes observed exclusively in the right hemisphere. Moreover, both the functional and structural assessments showed widespread changes in high-level association cortices, whereas changes in low-level sensory areas were less pronounced. The overlap between functional and structural measures suggests that enhanced connectivity aligns with structural changes due to crossmodal associations. These results illustrate that associations formed without explicit training or feedback can lead to functional and structural changes in the adult human brain, providing important implications for perception and neural plasticity in daily life situations.

**Supplementary Information:**

The online version contains supplementary material available at 10.1007/s00429-025-02951-3.

## Introduction

Associations play a crucial role in predicting and interpreting ambiguous sensory inputs, shaping our perception, and guiding our actions (Albright 2012). The process of associative learning involves establishing semantic connections between stimuli or behaviors based on their co-occurrence or similarity in outcomes. This type of learning can occur through classical conditioning, where a neutral stimulus becomes associated with a meaningful one, or operant conditioning, where behaviors are strengthened or weakened based on consequences (Hebb 1949; James 1890; Konorski 1967). Understanding these mechanisms is essential as they facilitate the acquisition and retention of new information, enabling organisms to effectively adapt sensory regularities and correspondences in external environment. Moreover, associations are tightly linked with neural plasticity, contributing to changes in neural connectivity and function (Albright 2012; Karim et al. 2021; McGann 2015).

The daily life experiences typically involve concurrent presentation of different sensory inputs, leading to systematic associations and correspondences between features encoded in different sensory modalities (Di Stefano and Spence 2023; Spence 2011). Consistent with this ecological observation, previous studies have shown that crossmodal associations can even be formed without explicit training via passive exposure to multisensory stimuli. For instance, Hidaka et al. (2011) showed that a rapid association (without any conditional reinforcement) between static tones and visual motion directions could be easily formed, and then the tones were able to drive perceived direction of visual motion (see also, Teramoto et al. 2010). Moreover, these audiovisual associations have been found to be selective for certain stimulus features, suggesting that they occur at the perceptual level rather than any decision level. Using motion types engaging pre-attentive visual processing, Kafaligonul and Oluk (2015) further revealed that these audiovisual associations and their effects on visual motion perception exist in absence of higher-order attentive tracking mechanisms, highlighting the automatic pre-attentive nature of sensory associations. Together with previous neurophysiological evidence on different visual features (Messinger et al. 2001; Schlack and Albright 2007), these psychophysical findings illustrate that associations are inherent nature of sensory and perceptual processing and may take place at different cortical areas. Despite these insights, we still have a limited understanding of how these audiovisual associations lead to functional and structural changes in the brain.

In this context, cortical thickness and resting-state functional connectivity measurements have been instrumental in elucidating experience-dependent structural and functional changes in the brain. Cortical thickness studies have shown that training in various tasks (Ditye et al. 2013; Draganski et al. 2004; Haier et al. 2009; Legault et al. 2019; Metzler-Baddeley et al. 2016; Wenger et al. 2012; Worschech et al. 2022) or the degree of visual experience (e.g., central vision loss, macular degeneration, congenital blindness) (Burge et al. 2016; Defenderfer et al. 2023; Park et al. 2009; Prins et al. 2016) can induce alterations in cortical thickness in the human brain. Specifically, prolonged engagement in perceptual, motor, and cognitive tasks has been linked to increases in cortical thickness in areas responsible for the processing of relevant information (e.g., Ditye et al. 2013; Draganski et al. 2004; Haier et al. 2009; Legault et al. 2019; Worschech et al. 2022). Previous studies also indicated that increased cortical thickness is positively correlated with the changes in behavioral performance (Ditye et al. 2013; Gilaie-Dotan et al. 2013; Kanai et al. 2010; for a review see Kanai and Rees 2011) and cortical thickness measures can even predict subsequent rate of perceptual learning in visual tasks (Frank et al. 2016). These studies underscore the well-established link between the brain's structural plasticity and learning across various domains. However, to date, there is no systematic investigation on structural changes elicited by exposure-based short-term audiovisual associations.

Resting-state functional connectivity has also been widely applied to track functional changes in response to experience-dependent plasticity and learning. Functional connectivity at rest provides a valuable index of the plasticity across brain regions involved in sensory processing (Guerra-Carrillo et al. 2014). Notably, this is supported by studies including motion perception (e.g., Sarabi et al. 2018; Urner et al. 2013) or using dynamic audiovisual paradigms in training (e.g., Powers et al. 2012). There is also evidence that even passive exposure to sensory stimuli can lead to changes in resting-state functional connectivity between auditory and visual cortices (Eckert et al. 2008; Zangenehpour and Zatorre 2010). Zangenehpoura and Zatorre (2010)) proposed that modulations of functional connectivity and activation shifts across sensory cortices may be driven by associations passively formed between auditory and visual stimuli. This hypothesis aligns with neuroanatomical studies revealing direct connections between primary auditory and primary visual cortices (e.g., Beer et al. 2011a, b; Clavagnier et al. 2004; Falchier et al. 2002; Garner and Keller 2022). A brief association phase to link static tones with motion direction may strengthen the crosstalk between auditory and visual cortical areas, leading to observed changes in motion perception. On the other side, previous research has also indicated that sounds can evoke imagery and activate the visual cortex through feedback from non-retinal resources (Petro et al. 2014; Vetter et al. 2014). This possibility highlights the strengthening of top-down feedback connections from multisensory association and/or non-sensory areas to visual cortices. Hence, the audiovisual associations may be mediated by changes in either feedforward crosstalk between primary sensory cortices or feedback connections between sensory and multisensory areas or higher order association areas. It is important to note that these possibilities are not mutually exclusive, and changes in both types of connections may jointly contribute to neural plasticity and the observed alterations in motion perception.

In the current study, we aimed to investigate the neuroplasticity associated with short-term audiovisual associations, focusing on both functional connectivity and cortical thickness. Building upon the established role of associations in shaping perceptual processes, our hypothesis posited that forming rapid associations between static tones and visual motion directions would lead to measurable alterations in both resting-state functional connectivity and cortical thickness within relevant brain regions. Unlike typical learning paradigms that require explicit training and feedback, we focused on association effects through passive exposure, allowing us to examine how crossmodal associations elicits plasticity without reinforcement. We utilized the random-dot motion paradigm originally developed by Hidaka et al. (2011), indicating that associations between the direction of coherent random dots and static tones can lead to strong changes in perceived direction of visual motion. In this paradigm, observers performed a perceptual task on visual motion while passively listening to sounds, with vision serving as the primary modality and audition as the secondary. The behavioral findings suggest that the information (i.e., the association acquired through passive exposure) provided by audition (secondary task-irrelevant stimulation) interferes and interacts with the motion processing which is primarily carried out by the visual system. Moreover, previous research has shown that this type of motion (i.e., random dots) and stimulation mainly engage early and mid-level levels of cortical processing (Britten et al. 1992; Ho and Giaschi 2009; Rina et al. 2022). In accordance with these findings, our primary neuroimaging focus was on occipital (V1–V3) and middle temporal (MT+/V5) areas. Specifically, we anticipated that the association established between the direction of visual motion and static tones would enhance the crosstalk between these visual areas and auditory cortices/audiovisual areas, as suggested by previous neuroanatomical and functional connectivity studies. Moreover, we expected connectivity changes to reflect enhanced neural integration and potentially increased cortical thickness in areas responsible for sensory processing. By exploring these dynamics, we aimed to contribute to a deeper understanding of how passive exposure to multisensory stimulation and crossmodal associations modulate brain structure and function, particularly in the context of audiovisual processing.

## Materials and methods

**Fig. 1:**
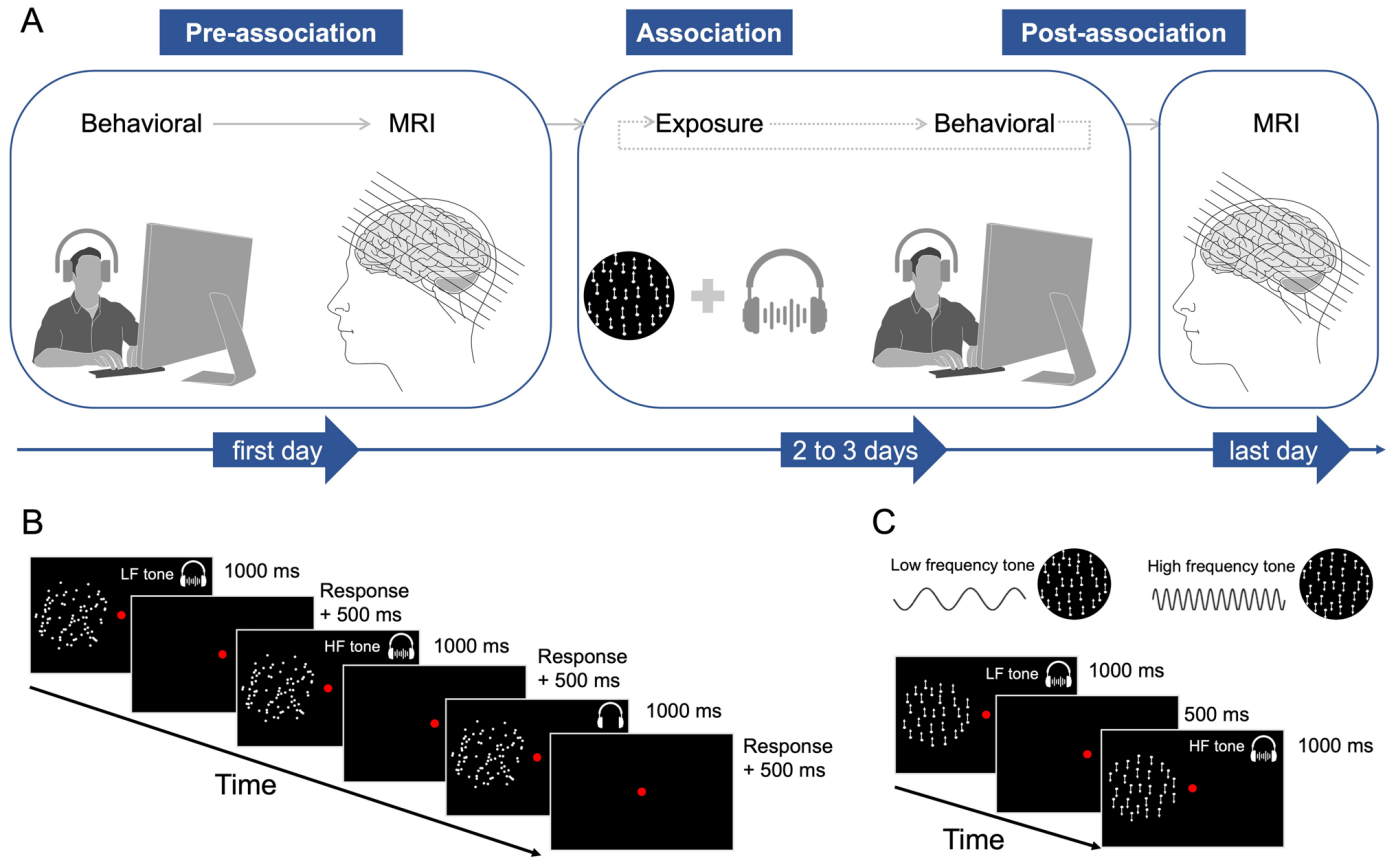
Experimental design **A** Illustration of experimental procedures taking place over the course of several days. Note that the last session of association (exposure and behavioral testing) took place on the last day. **B** Schematic depiction of the stimulation and timeline of events during a trial of behavioral measurement/assessment. Different levels of motion coherence and audiovisual conditions were used. **C** Stimulus representation during an association session. Each motion direction (100% motion coherence) was paired with static sounds of distinct frequencies. In this representation, the low and high frequency tones were paired with upward and downward motion directions, respectively

Similar to previous research (Hidaka et al. 2011; Kafaligonul and Oluk 2015), our experiment consisted of different phases conducted over the course of several days (Fig. 1a). Participants first took part in a behavioral measurement, followed by an fMRI session. In the following association phase, each participant completed a behavioral association session for 2–3 days. After each association phase, a behavioral assessment was also performed. For the last association phase, participants returned to the laboratory for a final behavioral association session and assessment, followed by a second fMRI session. Participants either underwent 2 or 3 association and behavioral sessions, as some showed the association effect by the second day, and others by the third day. Further information about each step and phase are provided in the following subsections.

### Participants

Fourteen participants took part in the study. The data from one participant were excluded due to technical problems during data collection. The data from the remaining 13 participants (nine females, age range: 21–27 years) were included in data analyses. The final sample size was comparable with functional neuroimaging studies including both audiovisual perceptual learning and resting-state connectivity analysis (e.g., Powers et al. 2012). All participants had normal or corrected-to-normal vision, and no history of neurological and psychiatric disorders by self-report. Before their participation, they were informed about experimental procedures and signed a consent form. All procedures were in accordance with the international guidelines (World Medical Association, 2013) and was approved by the local ethics committee at Faculty of Medicine, Ankara University. The participants were compensated for their time with monetary payment upon completing all steps of the study.

### Behavioral measurements and associations

#### Apparatus and stimuli

MATLAB version 7.12 (The MathWorks, Natick, MA) with PsychToolbox 3.0 extension (Brainard 1997; Pelli 1997) was used for stimulus generation, timing and behavioral data acquisition. Visual stimuli were presented on a 21"LCD computer monitor (NEC MultiSync 2190UXp; resolution: 1600 × 1200 pixels; refresh rate: 60 Hz). Auditory stimuli were introduced through a headset (Sennheiser HD 518). Participants binocularly viewed the monitor from a distance of approximately 57 cm, and were supported by a chin- and head-rest. Calibration of visual and auditory stimuli was carried out using a SpectroCAL photometer (Cambridge Research Systems, Rochester, Kent, UK) and a sound-level meter (SL-4010, Lutron Electronics, Taipei, TW). Behavioral responses were collected with a standard computer keyboard. The behavioral measurements and audiovisual association sessions were carried out in a dark and silent room.

A small red circle (0.2° diameter) at the center of the display served as a fixation target on a black background (0.27 cd/m^2^). Visual stimuli were random dot kinematograms (RDKs) consisting of gray dots (diameter = 0.2°, 75.80 cd/m^2^). The dots were presented at a fixed density of 3.5 dots/deg^2^, within an invisible 5° diameter circular aperture (Fig. 1b, c). The center of circular aperture was on the left visual field and 5° away from the fixation target. Visual stimuli were presented in the parafoveal and peripheral regions (2.5°–7.5° of visual angle) to stimulate motion-sensitive areas without requiring central fixation shifts. This approach targets naturally motion-sensitive regions in the parafoveal visual field (Smith et al. 1998), while also minimizing the risk of eye movements by keeping the stimuli within a fixed, stable area. Given that the stimuli were presented within a well-defined and stable visual field region (2.5° to 7.5°), and participants were instructed to maintain fixation on a centrally located target, any minor shifts in gaze would not significantly affect the results. A white noise algorithm was used to control the coherence of moving dots and determine the position of each dot in a random-dot frame (Britten et al. 1992; Pilly and Seitz 2009). According to this algorithm, the coherent motion was generated with a random selection of a percentage of dots in each random-dot frame to be replotted at a shifted location in a specific direction (upward or downward motion with a 5°/s speed). On the other hand, the remaining noise dots were shifted to a random location and hence, the noise dots had random speed and motion direction. It is important to note that the lifetime of coherent dots was probabilistic outcome of their proportion (i.e., motion coherence) in this algorithm. The lifetimes of coherent dots become short as their percentage decreases. However, the lifetimes get longer when the proportion of coherent dots increases. In behavioral test measurements, the motion coherence was varied by changing the percentage of dots (see Task and Procedure). For the association sessions, we used 100% coherent dots condition and all dots moved in a single direction and stayed on the display during the presentation of motion. A static tone burst (sampling rate: 44.1 kHz) was used as auditory stimulus. The tone burst was a rectangular windowed sinusoidal with either a low (500 Hz) or high (2 kHz) frequency, and delivered at a sound pressure level of 83 dB.

#### Task and procedure

The behavioral part of the study was conducted in three phases: pre-association test, association, and post-association test (Fig. 1a). In the pre- and post-association test phases, the task and procedure were exactly the same and the participants engaged in a motion direction discrimination task. On each trial, the fixation point was presented first for a variable duration (900–1000 ms). Then, the visual motion, along with auditory tones if introduced, was presented for 1 s. After the stimulus offset, participants were required to indicate whether the global dot motion was upwards or downwards by pressing up or down arrow keys on the keyboard, respectively (Fig. 1b). The participants were specifically instructed not to track any individual dot motion, but to maintain central fixation and attend to both visual motion and auditory stimuli while estimating the overall direction of global motion. For each trial, the motion coherence was pseudo-randomly selected from six different levels (5%, 15%, 30%, 45%, 60%, or 90%). The behavioral test phase included three audiovisual conditions (i.e., visual motion with low frequency tone, visual motion with high frequency tone, visual-only/no tone), six motion coherence levels and two motion directions (upward or downward). Each participant completed two blocks with 216 trials (2 blocks × 216 trials), leading to 12 trials per condition at the end of a behavioral measurement. The order of conditions was randomized within each block.

In the association phase, the timing of stimulus presentation was the same (Fig. 1c). However, instead of visual motion with varying coherence levels, only a 100% coherent motion was presented with concurrent static tones. New random dot displays were generated for each presentation. The direction of motion was paired with a tone of distinct frequency. For seven of the participants, the low-frequency tone was paired with an upward motion and the high-frequency tone with a downward motion and vice versa for the remaining six. For convenience, we refer to these audiovisual pairings according to the motion direction (e.g., upward tone: a tone paired with upward motion, downward tone: a tone paired with downward motion). Participants were instructed to attend to both visual motion and tones while fixating on the red circle at the center of screen. Each association session included 200 presentations (100 for each audiovisual pair), and the order of presentations was randomized within a session. A session lasted for a total of eight minutes. Right after an association session, a follow-up behavioral measurement was performed as described above. Participants did not receive feedback following their responses. This lack of feedback was a deliberate choice, as our aim was to evaluate the effects of audiovisual associations and sensory experiences without explicit reinforcement.

#### Behavioral data analysis

Behavioral data analysis was performed in R (v3.6.3) (R Core Team, 2021). Percentage of upward motion responses were calculated separately for each participant and experimental condition (i.e., pre- and post-association; visual-only, upward, and downward tones) and fitted with a cumulative normal function using the R package quickpsy (Linares and López-Moliner 2016) to compute coherence thresholds (i.e., the point of subjective equality; PSE) and slope values. The threshold and slope shifts for each test session (pre- and post-) of a participant were calculated by subtracting the threshold and slope values of the visual-only condition from those of audiovisual conditions (i.e., upward tone and downward tone). To evaluate the effect of audiovisual association training on perceived direction and discrimination, thresholds and slope shifts were separately analyzed via repeated-measures ANOVA with two main factors: test phase (pre- vs. post-association) and tone (upward vs. downward tone). Post-hoc t-tests were performed to further understand the source of main effects and interactions, and all p-values were FDR (false discovery rate) corrected (Benjamini and Hochberg 1995). Contrasts with *p* < 0.05 were considered as significant. Partial eta squared (${\eta }_{p}^{2}$) was calculated to measure the effect size, where a value of 0.01 was low, 0.04 moderate, and 0.1 high (Richardson 2011). To evaluate the effect of audiovisual association training on perceived direction and discrimination in the visual-only condition, PSE and slope values for the visual-only condition were contrasted between pre- vs post-association phases using t tests. Cohen’s d (*d*) was calculated to measure the effect size, where a value of 0.2 was low, 0.5 moderate, and 0.8 high.

### Magnetic resonance imaging

To reveal association induced changes in the human brain, MRI recordings were carried out before and after the behavioral association phase. MRI procedures were identical for pre- and post-association phases (Fig. 1a).

#### Data acquisition

All images were acquired by using a 3 T Siemens Magnetom Trio scanner with a 12-channel head-coil at the National Magnetic Resonance Research Center (UMRAM) at Bilkent University. To minimize head movements, a vacuum cushion and cushioned head stabilizers that reached cheeks from the sides of the head coil, were used. MR compatible headphones were utilized to eliminate potential sound artifacts that resulted from the MRI machine throughout data acquisition. High-resolution 3D anatomical scans were obtained with the following parameters: T1-weighted single turbo FLASH, TR/TE = 7982/3.68 ms, 1 mm isotropic voxels, FOV = 256 × 256 mm^2^, matrix size = 256 × 256 × 176. Resting-state functional scans were acquired in total darkness, during which observers were instructed to close their eyes, relax but not to sleep. The hemodynamic responses were acquired with a T2-weighted standard EPI sequence (TR/TE = 2000/35 ms, 3 mm isotropic voxels, slice thickness: 3 mm, FOV = 240 × 131.5 mm^2^, matrix size = 80 × 78 × 28), resulting in 360 volumes per scan. Each resting-state functional scan took 12 min.

#### Data preprocessing

*Anatomical data* T1-weighted (T1w) images were first corrected for intensity non-uniformity using Bias Field Correction Tool in statistical parametric mapping (SPM) version 12 (RRID: SCR_007037; Bazay et al. 2022). Then, they were processed using a recon-all processing stream which includes motion correction, skull-stripping, registration, segmentation, smoothing, and parcellation mapping. Standard retinotopic atlases to the individual’s FreeSurfer cortical reconstruction (RRID: SCR_001847) were created by using Anaconda 3, version 2020.11 (Benson et al. 2014; Benson and Winawer 2018).

*Functional data* fMRI data were preprocessed with CONN toolbox, version 17.f (RRID: SCR_009550; Whitfield-Gabrieli and Nieto-Castanon 2012) and SPM version 12 (RRID: SCR_007037; Bazay et al. 2022) using the standard preprocessing pipeline. Data were functionally realigned and unwrapped; slice-time corrected; structurally and functionally normalized and segmented into gray matter, white matter, and CSF tissue; flagged as potential outliers if framewise displacement exceeded 0.9 mm or if global BOLD signal changes were above five standard deviations; and smoothed, using a 6 mm Gaussian kernel (Whitfield-Gabrieli and Nieto-Castanon 2012). Subject-motion threshold was set to 1 mm, and both functional and structural data were resampled with 2 mm and 1 mm voxels, respectively. Artifacts were detected and entered into a linear regression as potential confounding effects (i.e., white matter, CSF, realignment, scrubbing, and the effect of rest) in order to remove any influence on the BOLD signal. After linear de-trending was performed, images were then band-pass filtered between 0.008–0.09 Hz and motion regressed to minimize the effect of motion and noise sources, as CONN removes temporal frequencies below or above these values by default in order to focus on slow-frequency fluctuations (Nieto-Castanon 2020).

#### Region of interest definitions

**Fig. 2:**
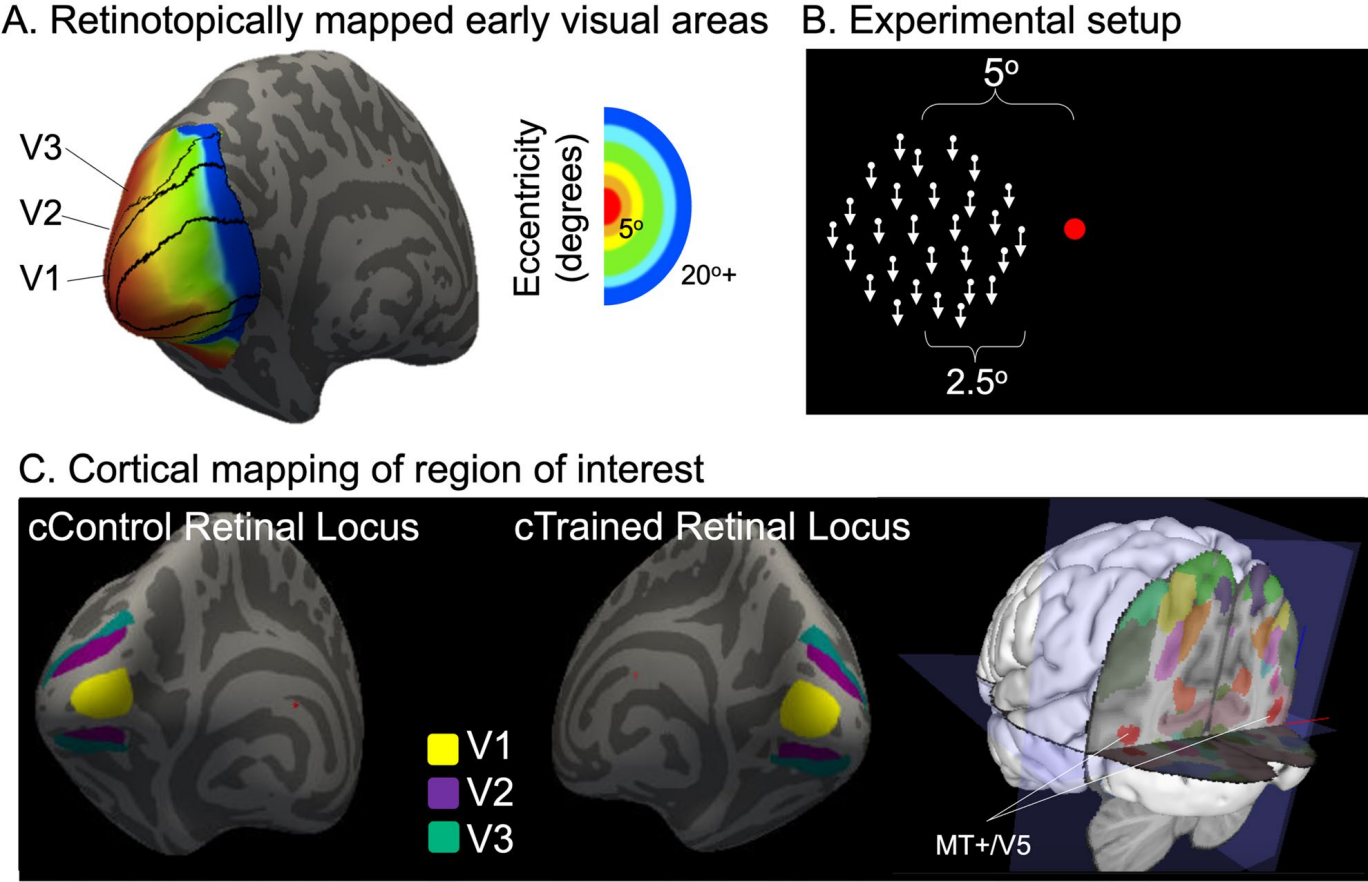
Identification of region of interests and their cortical projections. **A** Illustration of the anatomic atlases of eccentricity, polar angle and sigma mapping in early visual areas. Eccentricity is represented across the cortex with central vision at the occipital pole. **B** Schematic representation of the region of interests. During the behavioral assessments and association sessions, moving random dots were presented in the left visual field. Visual stimuli had a 2.5° radius and located 5° away from the fixation dot in the horizontal axis. **C** Cortical mappings of the regions of interest. Based on the anatomic atlases, we defined the cortical projections of the region of interests on the right and left visual fields in early visual areas (V1–V3) which corresponds to cControl and cTrained Retinal Loci at the left and right hemispheres, respectively. Bilateral MT+/V5 area was also defined as a region of interest

Regions of interests for functional connectivity analysis were defined in the bilateral visual field over early visual areas (V1–V3) based on Benson atlases (Benson et al. 2014) (Fig. 2a). A circular region of interest with 2.5 degrees of visual angle radius centered at 5° on the horizontal axis, corresponding to the trained and stimulated area in the left visual field (Fig. 2b), was identified for each participant and mapped onto the cortex by using cortical mapping method (Defenderfer et al. 2021) (Fig. 2c). Similarly, a circular region of interest with 2.5 degrees of visual angle radius centered at 5° on the horizontal axis, corresponding to the untrained area in the right visual field, was identified for each participant and mapped onto the cortex by using the same cortical mapping method (Defenderfer et al. 2021) (Fig. 2c). Trained and untrained areas in the left and right visual fields were first mapped on individuals’ native space and then converted into volumetric space by using the FSL FLIRT function. Cortical correspondence of the trained and untrained areas were named as cTrained Retinal Locus and cControl Retinal Locus, respectively. To be able to compare our results at the group level, we resampled ROIs (cTrained and cControl Retinal Loci) in MNI 2 mm standard space. Additionally, the bilateral MT+/V5 areas were defined based on Jülich histological atlas (Amunts et al. 2020) (Fig. 2c). Since the retinocortical mapping method (Defenderfer et al. 2021) applies only to early visual regions (V1–V3), the entire MT+/V5 region was used. Hence, cTrained and cControl Retinal Loci and bilateral MT+/V5 areas were used as seed ROIs in the functional connectivity analysis to investigate the connectivity changes with every other voxel in the brain, and in cortical thickness analysis to investigate the structural changes between pre- and post-association sessions.

#### Functional connectivity analysis

Functional connectivity measures were computed between a seed ROI and every other voxel in the brain (i.e., seed-to-voxel functional connectivity) by using the CONN toolbox v17.f (RRID: SCR_009550; Whitfield-Gabrieli and Nieto-Castanon 2012) to evaluate the effects of audiovisual association training. Eight major seed ROIs (cTrained Retinal Loci at V1, V2, and V3, cControl Retinal Loci at V1, V2, and V3, left MT +/V5, and right MT+/V5) were used to compute functional connectivity with every other voxel in the brain. cTrained and cControl Retinal Loci, and bilateral MT+/V5 were imported into the CONN toolbox for analysis. Based on the results obtained in the first-level analysis, a paired t-test was performed with a threshold set at *p* < 0.05 FDR-corrected to determine significantly different functional connections between pre-association and post-association assessments. A two-tailed t-test was used to compare post- and pre-association resting state scans to identify the effects of association training on functional connectivity. This allows for the detection of both increases and decreases in functional connectivity following training.

#### Cortical thickness assessment

Cortical thickness data was derived from T1-weighted MRI images. Cortical thickness was computed for 180 bilateral Glasser atlas regions (Glasser et al. 2016) and the cTrained (at V1, V2, V3) and cControl (at V1, V2, and V3) Retinal Loci using FreeSurfer 6.0.0's standard, automated cortical reconstruction pipeline on a Linux-based computing cluster. This resulted in 366 regions in total (i.e., 180 bilateral Glasser atlas regions, three cTrained Retinal Loci, and three cControl Retinal Loci). The processing steps include skull-stripping (Ségonne et al. 2004), Talairach transformation, subcortical gray and white matter segmentation (Fischl et al. 2002), intensity normalization (Sled et al. 1998), gray and white matter tessellation, topology correction (Fischl et al. 2001), and intensity gradient-based surface deformation to generate gray vs. white and gray vs. cerebrospinal fluid surface models (Dale et al. 1999; Fischl et al. 2001). The resulting surface models were then inflated and registered to a spherical surface atlas, allowing parcellation of cortical regions of interest. Finally, regional cortical thicknesses were computed by taking the mean of white-pial distance at all vertices within each parcellated region (Fischl and Dale 2000). Cortical thickness measurements within the Glasser atlas regions were extracted from anatomical scans that were acquired in both pre- and post-association assessments. Then, they were normalized by dividing to mean cortical thickness value for each participant’s pre- and post-association assessments.

#### Statistical analysis

Statistical analyses were performed using SPSS v29.0.2.0 (IBM SPSS Statistics, Armonk, NY, USA). Paired samples t-tests were used to compare within-subject effects of the audiovisual association training for cortical thickness and functional connectivity alterations. FDR procedure was applied to correct for false-positive inflation at multiple comparisons and FDR-corrected p values were used as a significance level to test our hypotheses. Paired sample t-test results were limited to FDR-corrected *p* < 0.05 values for both cortical thickness and functional connectivity comparisons.

## Results

We assessed the impact of audiovisual associations by analyzing the data collected before and after the association phase. Through a within-subject experimental design, we examined changes in behavior, cortical thickness, and functional connectivity to uncover the structural and functional plasticity resulting from multisensory experience.

### Participants could successfully associate visual and auditory stimuli

**Fig. 3:**
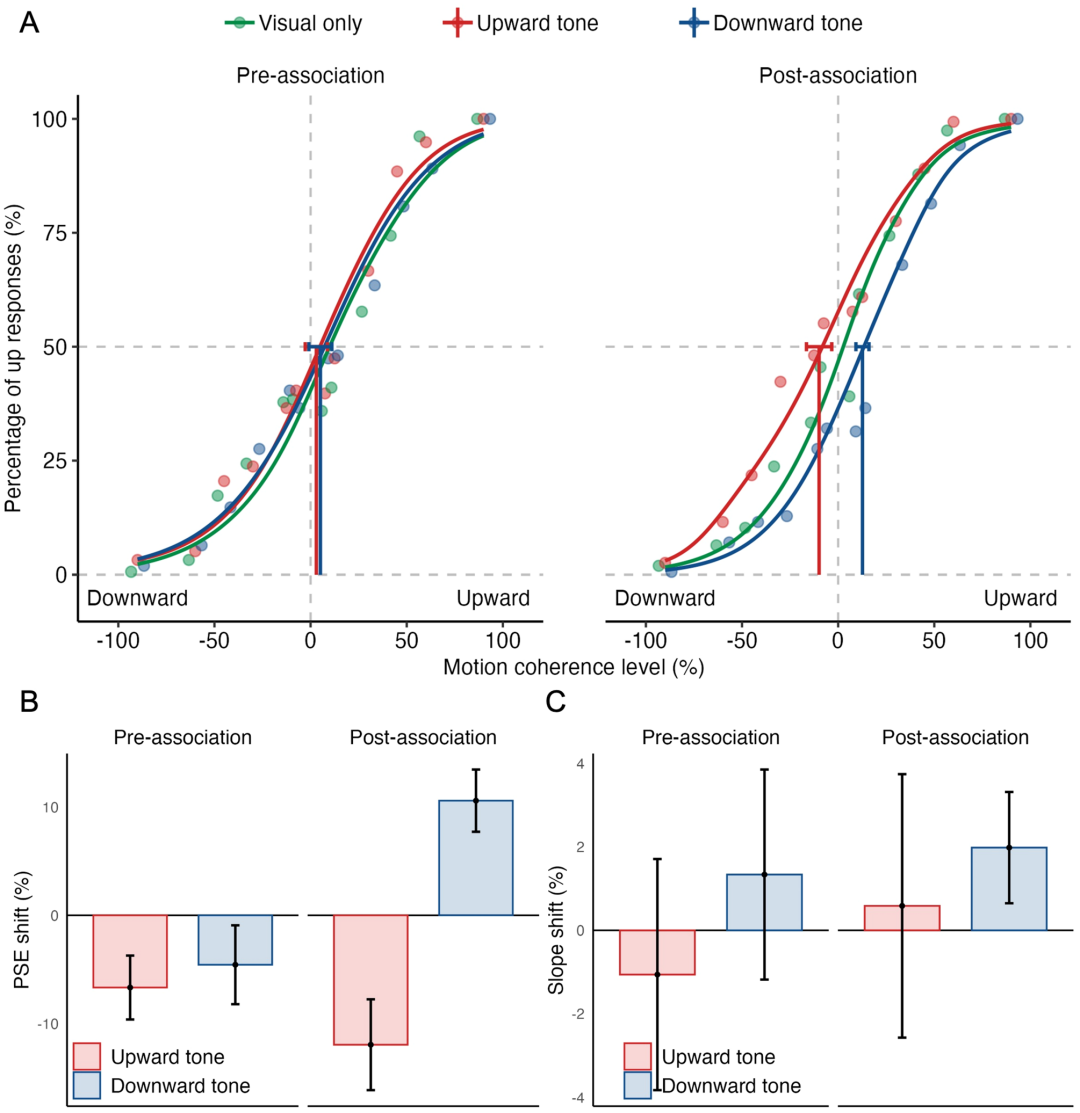
The effects of audiovisual associations on behavioral performance. **A** Group-averaged psychometric functions for pre- and post-association test phases. **B** The point of subjective equality (PSE) shifts as a function of the test phase, calculated by subtracting the PSE of visual-only condition from those of motion-sound conditions (i.e., upward and downward tone). **C** The slope shifts as a function of the test phase, calculated by subtracting the slope of visual-only condition from those of motion-sound conditions. Error bars denote the ± SEM

The effects of audiovisual associations on behavioral reports based on visual motion direction are shown in Fig. 3. The behavioral assessment on the first day was considered pre-association data, while the behavioral measurement right after the last association session of each participant were used as post-association data. The behavioral measurements on each day of the association phase are also provided in Figure S1 for each participant. Psychometric curves of visual-only, upward tone, and downward tone conditions as a function of motion coherence level are plotted for pre- and post-association test phases (Fig. 3a). Participants were not able to discriminate the direction of random dot motion when the coherence level was low. However, as the proportion of coherent dots increased (i.e., motion coherence level), participants reported motion direction in alignment with the dots'physical displacement, leading to improved performance, as depicted in Fig. 3a. In the pre-association test phase, the psychometric curves for the visual-only, upward tone, and downward tone conditions overlap, showing that static tones did not cause any change in the psychometric curves at this phase. After the paired presentation of motion directions and static tones during the association phase, however, the static tones significantly biased the reported direction of dot motion in favor of the exposed audiovisual pairing. In the post-association test phase, there was a noticeable shift in the curves: the upward and downward tone conditions shifted to the left and right, respectively, while the visual-only condition was relatively centered.

The analysis on the PSE differences between the visual-only and motion-sound conditions showed that both the main effect of tone condition (F(1, 12) = 12.80, *p* = 0.004, ${\eta }_{p}^{2}$ = 0.52) and the interaction between the tone and test phase (F(1, 12) = 7.65, *p* = 0.017, ${\eta }_{p}^{2}$= 0.39) were significant whereas the main effect of test phase was not significant (F(1, 12) = 2.63, *p* = 0.13, ${\eta }_{p}^{2}$ = 0.18) (Fig. 3b). Post hoc pairwise comparisons conducted on the interaction effect revealed a significant difference between upward and downward tone conditions for the post-test phase (MD = 0.23, SE = 0.047, t(12) = 3.44, *p* = 0.005). However, no significant difference was found between upward and downward tone conditions for the pre-test phase (MD = 0.02, SE = 0.051, t(12) = 0.74, *p* = 0.47). These results show that audiovisual association training effectively changed how participants reported the motion direction: the tone that was paired with upward motion (i.e., upward tone) led to an increase in reports of upward motion perception whereas the tone that was paired with downward motion (i.e., downward tone) led to an increase in reports of downward motion perception. The pairing of static tones with visual motion during the association phase could also potentially alter subsequent direction discrimination thresholds. Specifically, in the post-association test phase, it is also possible that static tones might significantly change direction discrimination performance compared to the visual-only condition. We explored this possibility by examining changes in slope values as depicted in Fig. 3c. However, a two-way repeated measures ANOVA (with test phase and tone condition as factors) did not yield any significant main effects (test phase: F(1, 12) = 0.13, *p* = 0.73, ${\eta }_{p}^{2}$ = 0.011, tone condition: F(1, 12) = 1.12, *p* = 0.31, ${\eta }_{p}^{2}$ = 0.086) or a two-way interaction (F(1, 12) = 0.092, *p* = 0.77, ${\eta }_{p}^{2}$ = 0.008). Overall, our findings did not indicate consistent changes in slope values specific to tone conditions or improvements/decline in direction discrimination performance due to audiovisual associations.

To assess whether association learning or/and behavioral training related changes occurred in trials without static sounds (i.e., visual-only), PSE and slope values for the visual only condition were contrasted between pre- vs post-association phases (Figure S2). A paired t-test comparing PSE values in visual-only condition between the pre- and post-association phases found a significant decrease in PSE values from pre- to post-association, MD = 0.076, t(12) = 2.74, *p* = 0.018, d = 0.76, 95% CI [0.016, 0.14]. A paired t-test for slope values in visual only condition yielded no significant difference between the pre- and post-association phases, MD = 0.06, t(12) = 1.68, *p* = 0.12, d = 0.47, 95% CI [− 0.018, 0.14]. These findings indicate that in the visual-only condition, observers were not biased in favor of the exposed associations, and their motion discrimination sensitivity did not improve from pre- to post-association phase.

### Functional connectivity changed between early visual regions and association areas

**Fig. 4:**
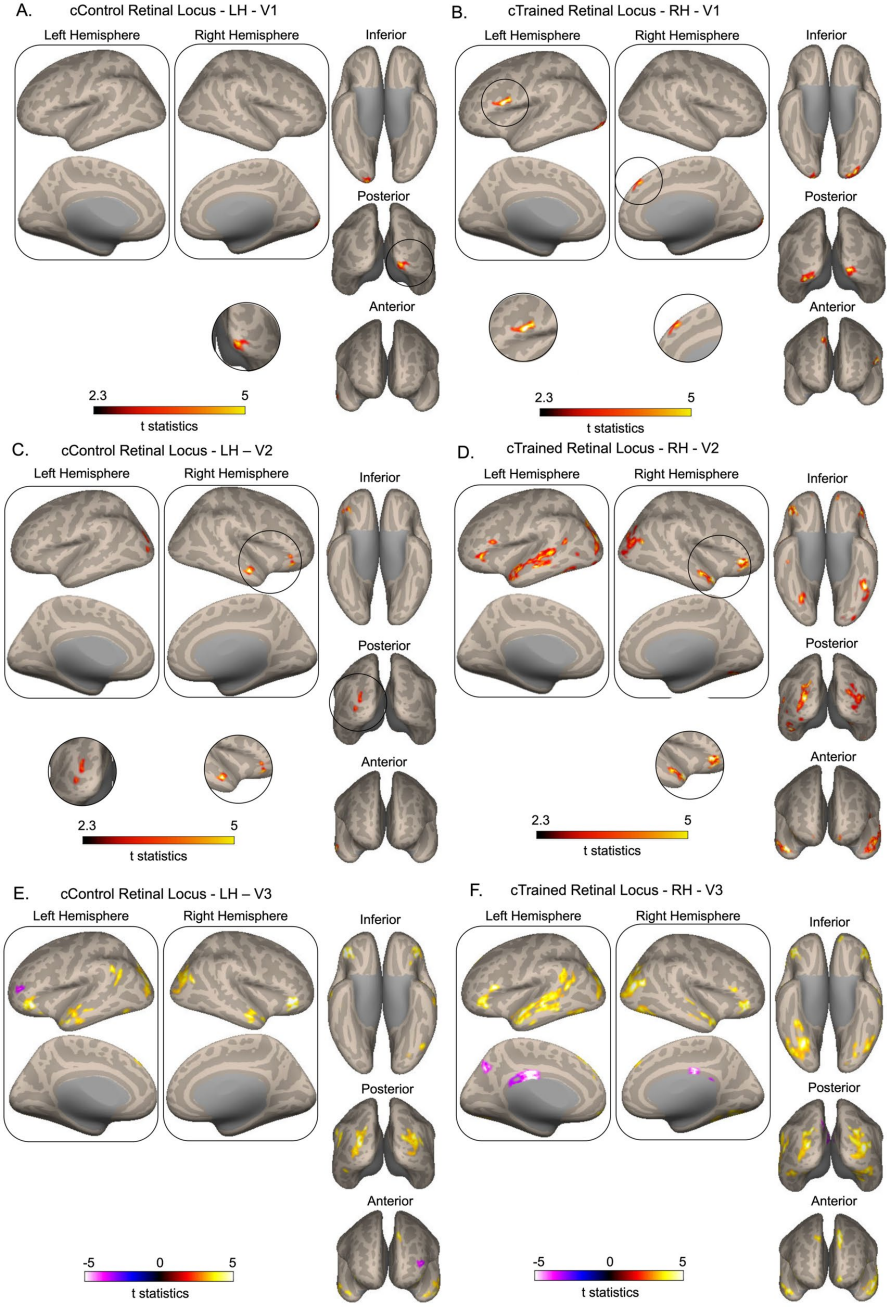
Functional connectivity changes between the cortical correspondence of control (cControl Retinal Loci) and trained (cTrained Retinal Loci) seed areas in early visual areas (V1, V2, V3) and the whole brain (**A**–**F**). Seed-to-voxel functional connectivity changes occurred across a wider region for cTrained Retinal Loci than cControl Retinal loci. Paired sample t-test results were limited to FDR-corrected *p* < 0.05. T statistics results were projected on standard space cortical surface. Dark red-bright yellow color bar scale (in panels **A**–**D**) indicates t statistics between 2.3 and 5. White-bright yellow color bar scale (in panels **E**–**F**) indicates t statistics between −5 and 5. Brighter colors represent higher t values. Clusters that are difficult to see have been enlarged and displayed below the main brain images for better visibility

Seed-to-voxel functional connectivity analyses examined the connectivity of cortical correspondence of both trained and control seed areas in early visual areas (V1, V2, V3) with the whole brain (Table 1. and Fig. 4). The analysis revealed significant changes mostly in post > pre direction, with connectivity increasing from the pre- to post-association phase whereas post < pre changes, with connectivity decreasing from the pre- to post-association phase, were few. More importantly, functional connectivity changes were notably larger for cTrained Retinal Loci in the right hemisphere than for cControl Retinal Loci in the left hemisphere in all early visual areas (V1, V2, V3).

**Table 1:** Functional connectivity changes between seed regions and whole-brain voxels following association

Seed region	Cortical areas	MNI coordinates	Cluster size	*t*(12) = ± 3.05, *p* values
*Post > pre*				
cTRL in V1, RH	Occipital pole, RH	+ 10 − 98 − 2	259	0.002
	Occipital pole, LH	− 30 − 92 − 18	246	0.003
	Inferior frontal gyrus, pars opercularis, LH	− 50 + 8 + 16	244	0.004
	Superior frontal gyrus, RH	+ 10 + 38 + 44	137	0.026
cCRL in V1, LH	Occipital pole, RH	+ 10 − 98 − 2	181	0.019
cTRL in V2, RH	Middle temporal gyrus posterior division, LH	− 58 + 22 + 4	1686	< 0.001
	Temporal pole, RH	+ 48 + 14 − 20	844	< 0.001
	Lateral occipital cortex superior division, LH	− 30 − 80 − 4	689	< 0.001
	Lateral occipital cortex superior division, RH	+ 28 − 76 + 10	604	< 0.001
	Inferior frontal gyrus, LH	− 42 − 46 − 16	351	0.0002
	Cingulate gyrus posterior division	− 2 − 20 + 28	154	0.003
	Precuneus cortex, LH	− 8 − 64 + 36	188	0.003
	Frontal pole, LH	+ 6 + 68 − 18	130	0.022
cCRL in V2, LH	Lateral occipital cortex, superior division, LH	− 26 − 84 + 4	239	0.002
	Middle temporal gyrus, RH	+ 46 − 10 − 24	154	0.019
	Frontal orbital cortex, RH	+ 40 + 32 − 16	146	0.019
cTRL in V3, RH	Middle temporal gyrus, LH	− 59 + 24 + 18	3694	< 0.001
	Lateral occipital cortex RH	+ 34 − 72 + 12	1552	< 0.001
	Frontal pole, LH	− 10 + 44 + 44	504	< 0.001
	Temporal pole, RH	+ 48 + 14 − 20	394	< 0.001
	Frontal pole, RH	+ 52 + 44 − 6	374	< 0.001
	Frontal pole, LH	− 10 + 64 − 18	179	0.004
	Temporal occipital fusiform, RH	+ 32 − 40 − 22	130	0.019
	Inferior frontal gyrus, RH	+ 62 + 18 + 24	125	0.019
cCRL in V3, LH	Frontal orbital cortex, LH	− 48 + 48 − 12	920	< 0.001
	Lateral occipital cortex superior division, LH	− 30 − 80 + 14	698	< 0.001
	Lateral occipital cortex superior division, RH	+ 36 − 66 + 18	591	< 0.001
	Frontal pole, RH	+ 44 + 32 − 8	520	< 0.001
	Middle temporal gyrus anterior division, RH	+ 62 − 6 − 16	243	0.0006
	Angular gyrus, LH	− 44 − 50 + 26	155	0.009
	Frontal pole, LH	− 8 + 44 + 48	116	0.030
MT +/V5, RH	Postcentral gyrus, LH	− 40 − 38 + 56	197	0.005
	Postcentral Gyrus, RH	+ 26 − 20 + 42	180	0.005
MT +/V5, LH	Lateral occipital cortex, RH	+ 38 − 74 + 6	547	< 0.001
	Superior temporal gyrus, RH	+ 70 − 26 + 16	142	0.015
	Occipital pole, LH	− 6 − 104 + 12	141	0.015
*post* < *pre*				
cTRL in V3, RH	Cingulate gyrus	− 2 − 20 + 30	354	< 0.001
	Precuneus cortex	− 8 − 66 + 36	146	0.011
cCRL in V3, LH	Frontal Pole, LH	− 34 + 56 + 16	116	0.030

In V1, functional connectivity changes were notably larger for cTrained Retinal Locus than for cControl Retinal Locus. Specifically, cTrained Retinal Locus in right V1 exhibited stronger and more widespread connectivity increases, involving the right and left occipital poles, the left inferior frontal gyrus (pars opercularis), and the right superior frontal gyrus (Fig. 4b). In contrast, cControl Retinal Locus in left V1 showed a more limited increase, with significant connectivity enhancement observed only with the right occipital pole with a smaller region (Fig. 4a).

In V2, functional connectivity changes were larger for cTrained Retinal Locus than for cControl Retinal Locus. cTrained Retinal Locus in right V2 displayed more widespread connectivity increases, particularly with the left middle temporal gyrus (posterior division), right temporal pole, bilateral lateral occipital cortex (superior division), left inferior frontal gyrus, posterior cingulate gyrus, left precuneus, and the left frontal pole (Fig. 4d). On the other hand, cControl Retinal Locus in left V2 exhibited more restricted connectivity changes, showing significant increases with the left lateral occipital cortex (superior division), right middle temporal gyrus, and the right frontal orbital cortex (Fig. 4c).

In V3, connectivity increases again favored the trained region, with cTrained Retinal Locus in right V3 showing widespread connectivity enhancements involving the left middle temporal gyrus, right lateral occipital cortex, bilateral frontal pole, right temporal pole, right temporal occipital fusiform gyrus, and the right inferior frontal gyrus (Fig. 4f). In contrast, cControl Retinal Locus in left V3 showed fewer and less extensive connectivity increases, with enhanced connectivity observed in the left frontal orbital cortex, bilateral lateral occipital cortex (superior division), bilateral frontal pole, right middle temporal gyrus (anterior division), and left angular gyrus (Fig. 4e). In V3, connectivity decreases occurred as well. cTrained Retinal Locus in right V3 showed reduced connectivity with cingulate gyrus and precuneus cortex (Fig. 4f) while cControl Retinal Locus in left V3 showed reduced connectivity with a cluster in the left frontal pole (Fig. 4e).

**Fig. 5:**
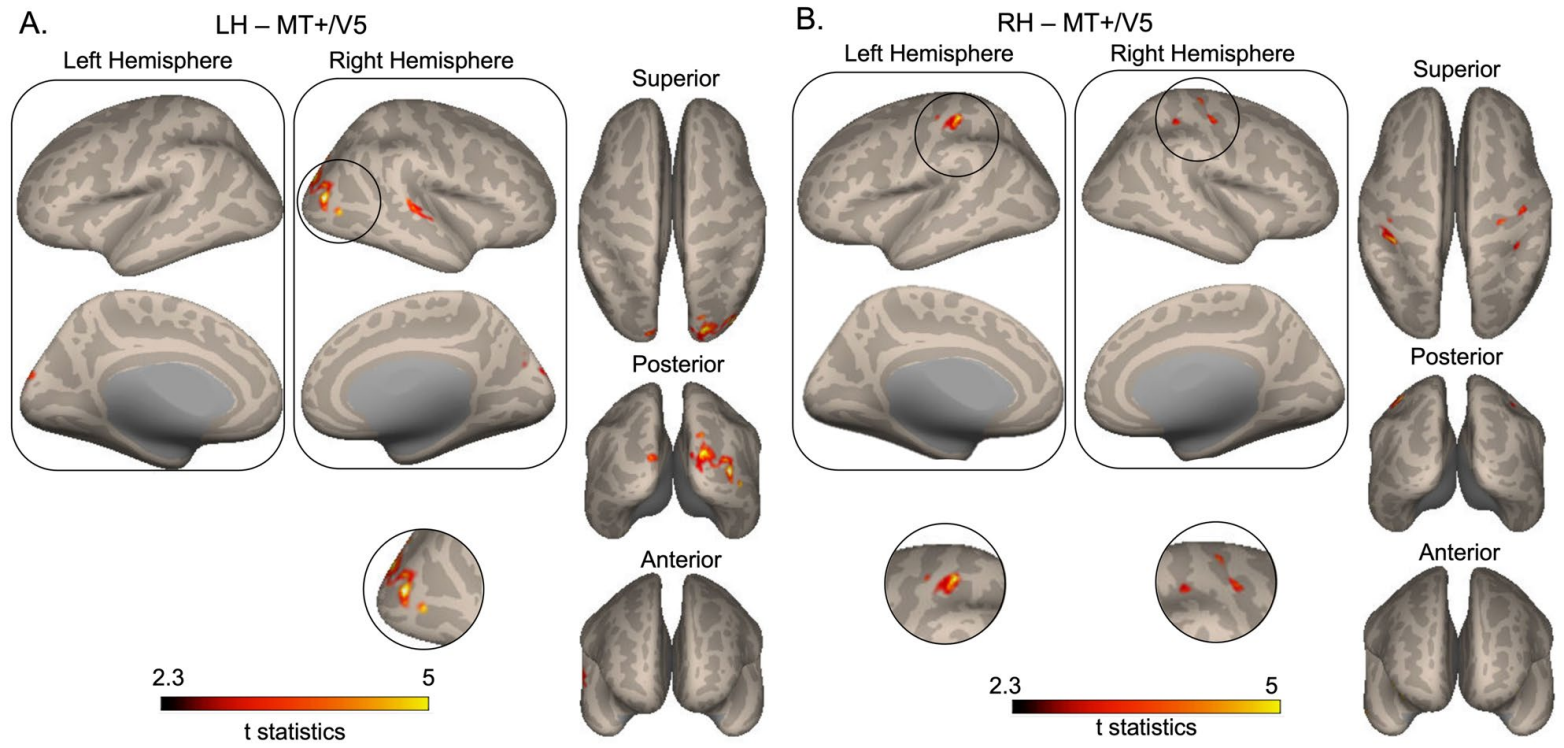
Functional connectivity changes between bilateral MT+/V5 and the whole brain. **A** Functional connectivity increased between the seed left MT +/V5 and the right lateral occipital cortex, right superior temporal gyrus, and left occipital pole. **B** Functional connectivity increased between the seed right MT+/V5 and bilateral postcentral gyrus. Paired sample t-test results were limited to FDR-corrected *p* < 0.05. T statistics results were projected on standard space cortical surface. Dark red-bright yellow color bar scale indicates t statistics between 2.3 and 5. Brighter colors represent higher t values. Clusters that are difficult to see have been enlarged and displayed below the main brain images for better visibility

Seed-to-voxel functional connectivity analyses were also conducted between bilateral MT+/V5 areas and the whole brain (Table 1. and Fig. 5). Functional connectivity between right MT+/V5 area and bilateral postcentral gyrus was increased in post-association compared to pre-association (Fig. 5b). In addition, functional connectivity between left MT+/V5 area and right lateral occipital cortex, right superior temporal gyrus, and left occipital pole was increased in post-association compared to pre-association (Fig. 5a).

### Cortical thickness alterations after association phase

**Fig. 6:**
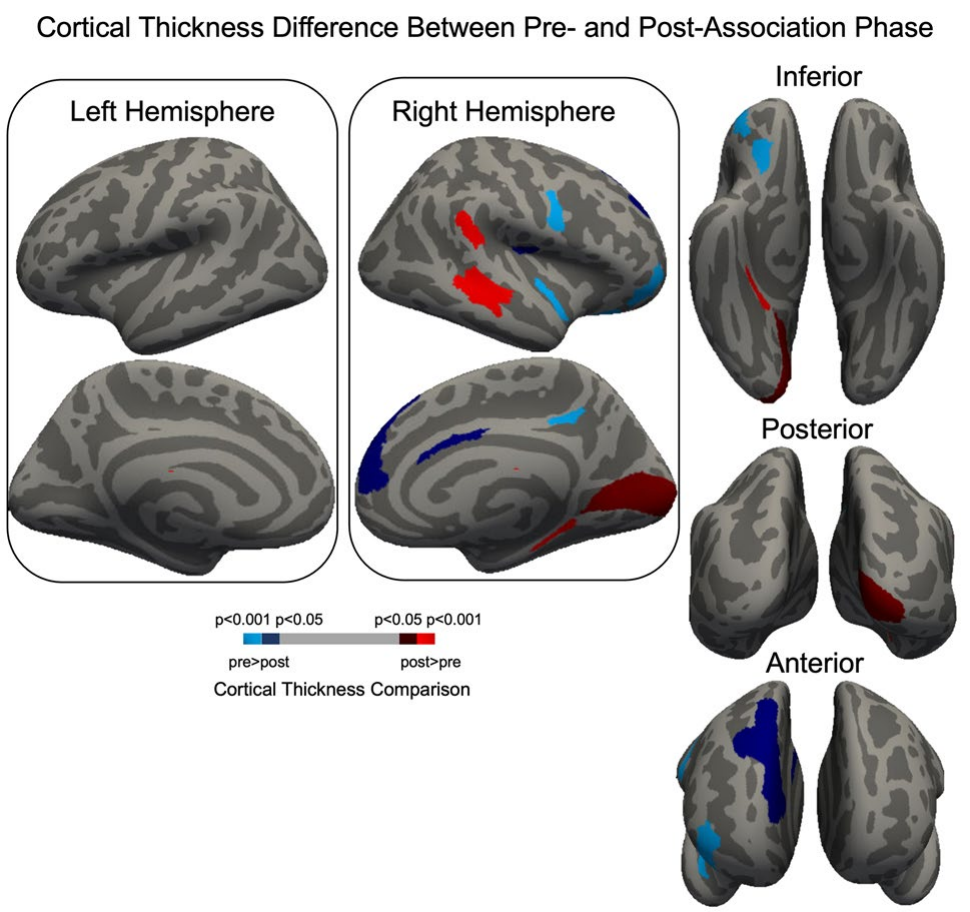
Whole-brain cortical thickness changes from pre- to post-association phase. Cortical thickness changes occurred exclusively in the right hemisphere. Paired sample t-test results were limited to FDR-corrected *p* < 0.05. Statistically significant cortical thickness results were projected on a standard space cortical surface. Red color bar scale indicates increased cortical thickness whereas blue color bar scale represents decreased cortical thickness after the association phase. Bright colors represent more significant results

Cortical thickness changes occurred exclusively in the right hemisphere, with both increases and decreases observed across multiple regions (Fig. 6). The results of FDR-corrected paired sample t-tests are summarized in Table 2.. Post-association cortical thickening was observed in the right primary visual cortex. In the temporal cortex, cortical thickness increased in the right PeriSylvian language area, right parahippocampal area 1, and both the right dorsal and ventral posterior superior temporal sulcus. A cortical thinning following association phase was found in multiple frontal and cingulate areas. In the frontal lobe, significant decreases were observed in the right ventral premotor area, area 9 m in and area 8B lateral in the right hemisphere, right posterior area 9, and right anterior area 47r. In the cingulate cortex, cortical thickness decreased in area 33 prime and area 31a in the right hemisphere. Additionally, reductions were observed in the right para-insular area and area 13 l in the right hemisphere. These results overall provide evidence that exposure-based associations and sensory experiences lead to structural plasticity in the adult brain.

**Table 2:** Whole-brain cortical thickness changes from pre- to post-association phase

Cortical region	Mean difference	CI lower -CI upper	t value (df = 12)	Adjusted p value
*Post* > *pre*				
Primary visual cortex, RH	0.027	0.01, 0.04	3.98	0.046
PeriSylvian language area, RH	0.095	0.06, 0.13	5.73	0.012
Parahippocampal area 1, RH	0.114	0.07, 0.16	5.23	0.015
Superior temporal sulcus dorsal posterior, RH	0.083	0.04, 0.11	4.89	0.018
Superior temporal sulcus ventral posterior, RH	0.133	0.09, 0.18	6.54	0.005
*Post* < *pre*				
Ventral premotor area, RH	− 0.142	− 0.20, − 0.08	− 5.03	0.018
Area 33 prime, RH	− 0.118	− 0.18, − 0.06	− 4.24	0.039
Area 9 m, RH	− 0.058	− 0.09, − 0.03	− 4.23	0.039
Area 8B lateral, RH	− 0.089	− 0.14, − 0.04	− 3.97	0.046
Area 9 posterior, RH	− 0.073	− 0.11, − 0.03	− 4.05	0.046
Area anterior 47r, RH	− 0.092	− 0.13, − 0.05	− 4.77	0.020
Area 13 l, RH	− 0.165	− 0.20, − 0.13	− 9.34	< 0.001
Area OP2-3 VS, RH	− 0.078	− 0.12, − 0.04	− 3.99	0.046
Area 31a, RH	− 0.164	− 0.23, − 0.10	− 5.50	0.012
Para insular area, RH	− 0.136	− 0.20, − 0.07	− 4.84	0.018

*Note.* The voxel threshold was *p* <.05. RH: right hemisphere. CI: confidence interval. Cortical regions are labelled according to the glasser atlas

## Discussion

Our findings provide compelling evidence that passive exposure to audiovisual associations induces both behavioral shifts and neural plasticity in the adult brain. Behaviorally, the reports based on coherent motion perception were significantly influenced by audiovisual associations, as evidenced by systematic shifts in psychometric functions following exposure. More importantly, resting-state fMRI revealed widespread increases in connectivity between the cortical representation of the trained region in early visual areas (V1–V3) and higher-order occipital, temporal, and frontal regions, whereas connectivity changes for the untrained region were markedly smaller. Additionally, area MT+ showed increased connectivity with occipital, temporal, and somatosensory areas, further supporting experience-dependent network reorganization. Interestingly, cortical thickness changes were observed exclusively in the right hemisphere, primarily in higher-order association regions, with only one significant change in primary visual cortex (V1). Together, these findings demonstrate that passive exposure to audiovisual pairings induces plasticity beyond early sensory areas, extending to large-scale functional networks and structural reorganization in higher-order cortical regions.

### Audiovisual associations influence behavioral reports of motion direction

Our behavioral results showed that audiovisual associations can lead to significant changes in the reports on visual motion direction. Specifically, as indicated by significant changes in coherence thresholds, we observed a notable shift in psychometric curves, suggesting a strong influence of associated static tones on perceived motion direction. These findings overall confirm that passive exposure to multisensory stimuli can lead to associative influences and thus cause major changes in subsequent perceptual judgements, particularly when the input to the visual system becomes ambiguous (Albright 2012; Hidaka et al. 2011; Kafaligonul and Oluk 2015; Teramoto et al. 2010).

A potential concern regarding these findings is whether the behavioral shifts reflect decisional biases rather than perceptual changes. In our study, the absence of systematic changes in the slope values across tone conditions provides some evidence against decisional biases as the sole explanation. If participants had relied purely on auditory cues while disregarding visual motion, we would expect changes in psychometric curves (e.g., changes in slope values) in the post-association test phase, indicating altered sensitivity to visual motion. However, our findings do not support this prediction.

That said, it remains possible participants adopted response strategies based on learned audiovisual associations, leading to shifts in their judgments without directly altering sensory processing. Prior studies have shown that response biases can shift the central tendency of psychometric curves (i.e., PSE) without affecting their slope, highlighting the importance of distinguishing between perceptual and decisional influences (García-Pérez and Alcalá-Quintana 2013; Morgan et al. 2012). One approach to distinguish these effects is to assess whether the learned influence on perception decays over time, as perceptual effects typically diminish, whereas decisional biases are more persistent (Winawer et al. 2010). While our design did not include delays between association and test phases to examine this, future studies could explore this possibility.

Previous research using similar experimental designs and stimuli has shown that audiovisual associations are selectively influenced by low-level stimulus features (e.g., visual field, sound frequency) and depend on the specific motion type used during the association and test phases, suggesting perceptual changes (Kafaligonul and Oluk 2015; Kobayashi et al. 2012a, 2012b). Our analyses further support this, showing that connectivity changes were substantial in the trained visual hemifield compared to the untrained hemifield (see Fig. 4). This suggests that the effects of learned associations were mostly localized to the region where exposure occurred, implying that the changes are likely driven by alterations in perceptual processing mechanisms (Hidaka et al. 2011; Kafaligonul and Oluk 2015; Kobayashi et al. 2012a, b). These findings suggest that perceptual changes likely contribute to the observed effects, even if we cannot completely rule out the contribution of decisional biases.

Overall, our findings, along with prior research on crossmodal influences on vision (Altieri et al. 2015; Gibson and Maunsell 1997; Gonzalo et al. 2000; Shams and Seitz 2008; Tanabe et al. 2005; Zangenehpour and Zatorre 2010) highlight the flexibility of sensory processing mechanisms and their role in adaptive behavior. Specifically, the current results suggest that the brain is capable of forming coherent perceptual experiences by monitoring information across sensory modalities, even without explicit training or reinforcement. This crossmodal adaptability may be a fundamental aspect of sensory processing, supporting the integration of passively learned associations that enhance perception, particularly under ambiguous conditions (Albright 2012).

### Audiovisual associations influence resting-state functional connectivity

Our seed-to-voxel analyses revealed that exposure-based audiovisual associations may lead to widespread increases in functional connectivity, with these changes being markedly more pronounced for the cortical representation of the trained region in early visual cortices compared to the untrained (control) region. Specifically, the trained seed regions—mapped to the right hemisphere’s V1, V2, and V3 (reflecting stimulation in the left visual field)—showed robust connectivity enhancements with a variety of higher-order regions including the lateral occipital cortex, middle temporal gyrus, frontal pole, temporal pole, inferior frontal gyrus, and occipital fusiform gyrus and a low-level sensory region (i.e., occipital pole). In contrast, the untrained seed regions, located in the left hemisphere, exhibited significantly smaller connectivity changes, highlighting the spatial specificity of the plasticity induced by the audiovisual associations. The seed-to-voxel analyses also revealed decreases in functional connectivity after association phase, but these changes were much less prominent, being found only at three small clusters at cingulate cortex, precunes cortex for the trained V3 and frontal pole for the control V3 region. Finally, our secondary analysis using bilateral MT+/V5 as seed regions revealed increased connectivity with primary sensory regions such as the occipital pole and postcentral gyrus, as well as with higher-order areas including the lateral occipital cortex and superior temporal gyrus.

We found that both the size and the number of clusters for functional connections were more pronounced for high level regions (e.g., middle temporal gyrus, lateral occipital cortex) than for low level regions (e.g., occipital pole) (see Table 1.). Although the current experimental design and analyses do not directly identify the direction of information flow between these regions and reveal the contributions of feedforward and feedback connections, previous multisensory and learning studies emphasize top-down mechanisms. For example, models of brain function, such as predictive coding, posit that higher-level regions generate predictions about sensory input and send these predictions back to lower-level areas to be compared with actual input (Clark 2013; de-Wit et al. 2010; Friston 2005; Rao and Ballard 1999). In our study, the increased connectivity between seed areas and high-level brain regions may represent a refinement of these feedback loops driven by audiovisual experience (Petro et al. 2017; Vetter et al. 2014). Moreover, the involvement of regions such as the frontal pole, temporal pole, and inferior frontal gyrus, which are known for their roles in higher-order cognitive functions, suggests that feedback processes are likely enhanced. These regions typically exert top-down control rather than serving purely as relay stations for feedforward sensory information (Koechlin et al. 2003; Miller and Cohen 2001). Furthermore, perceptual learning similarly involves a strengthening of top-down feedback mechanisms to refine sensory processing (Ahissar and Hochstein 2004). When high-level regions become more functionally connected to sensory areas after training, these regions are more effectively exerting control and modulation, a hallmark of top-down feedback.

We observed more substantial functional connectivity changes for cTrained and cControl seed regions in V2 (eleven clusters across eight different functional regions) and V3 (18 clusters across ten different regions), with none of these connectivity changes involving primary sensory regions. By contrast, cTrained and cControl seed regions in V1 showed connectivity changes in only five clusters, and MT+/V5 in five clusters, yet these changes included connections with primary sensory regions, such as the occipital pole and postcentral gyrus. This pattern—where connectivity changes for V2 and V3 predominantly involved higher-order association areas—underscores the significant role of these regions in audiovisual associations, suggesting that they may be particularly responsive to crossmodal interactions. Notably, this finding aligns with the reverse hierarchy theory of perceptual experiences, which proposes that practice-induced changes occur earlier and more robustly in higher cortical areas than in primary sensory areas, making these higher-level changes more detectable (Ahissar and Hochstein 2004). The connectivity patterns we observed thus provide evidence that even associations without explicit feedback can rely more on higher-level cortical areas, with changes in these regions occurring more rapidly and extensively than in lower-level sensory areas.

Our analysis on resting-state recordings did not reveal any connectivity changes (i.e., feedforward crosstalk) between primary auditory and visual cortices that are traditionally thought to be'sensory-specific'. This may suggest that passive exposure to audiovisual associations over a few days might not be sufficient to induce detectable changes in resting-state connectivity between primary sensory areas. On the other hand, prior research has demonstrated that changes in audiovisual associations can also emerge from interactions at early sensory levels, particularly when auditory and visual stimuli are both temporally and spatially congruent (Beer et al. 2011a, b; Garner and Keller 2022). A key distinction between our study and these previous studies is that our audiovisual pairings were temporally aligned but not necessarily spatially congruent, which may have influenced the degree to which early sensory interactions were engaged. Additionally, studies reporting plasticity at lower-level sensory areas typically involve prolonged or repeated training, whereas our paradigm included only a few days of passive exposure.

Interestingly, we found strengthened connections between low-level seed regions (V1 and MT+/V5) and primary sensory regions like the occipital pole and postcentral gyrus. The association phase in our design included both an association session (i.e., consecutive presentations of audiovisual pairings) and a behavioral measurement. After each association session, the participants performed a direction discrimination task in tone and no tone (visual-only) conditions to measure coherence thresholds and direction discrimination performance. It is conceivable that the connectivity increase between low-level visual areas may be driven by unisensory experience (e.g., no tone condition) and training (direction discrimination of visual motion in all conditions) during behavioral measurements. It is also possible that the sensory experience and training acquired during behavioral measurements might contribute to changes in resting-state connectivity across other regions mentioned above.

Overall, while it is possible that the connectivity increases in resting-state include components of heightened feedforward processing, the substantial involvement of high-level association areas in contrast to nonsignificant changes in connectivity between primary auditory and visual areas highlights an important role of top-down feedback mechanisms in crossmodal associations and correspondence. The enhancements of functional connectivity likely reflect the brain's adaptation to better integrate and interpret multisensory information through improved feedback control (e.g., Rohe et al. 2019).

### Structural plasticity following audiovisual associations

In addition to the robust functional connectivity changes observed in the trained regions (at the right V1, V2, and V3), our cortical thickness analyses revealed a parallel pattern of structural plasticity that was exclusively localized to the right hemisphere. Specifically, post-association cortical thickening was observed in the right primary visual cortex, as well as in several temporal regions, including the right PeriSylvian language area, right parahippocampal area 1, and both the right dorsal and ventral posterior superior temporal sulcus. Conversely, significant cortical thinning was detected in multiple frontal areas—namely the right ventral premotor area, areas 9 m and 8B lateral, the right posterior area 9, and right anterior area 47r—as well as in cingulate regions (areas 33 prime and 31a) and the para-insular area/area 13 l.

This pattern of structural changes resembles the functional connectivity results, where the trained regions in right early visual cortices exhibited widespread increases in connectivity with higher-order association areas—including frontal, temporal, and occipital regions—while the untrained regions in the left hemisphere showed markedly smaller connectivity changes. Notably, most of the significant cortical thickness alterations were located in higher-order regions, with only the primary visual cortex among the low-level sensory areas showing a significant increase. This convergence suggests that passively learned audiovisual associations lead to coordinated functional and structural reorganization, potentially reflecting enhanced top-down modulation and the refinement of feedback mechanisms across the cortical hierarchy.

The increased cortical thickness in occipital (i.e., V1) and temporal regions—areas known to support multisensory integration and memory (Albright 2012; Beauchamp 2005; Simons and Spiers 2003)—parallels the enhanced functional connectivity with the low (i.e., occipital pole) and higher-level areas. This expansion in cortical thickness suggests a structural basis for enhanced integrative capacities following associative learning (May 2011). Together, these findings point to a coordinated structural and functional reorganization that may underlie more effective top-down modulation. Conversely, the cortical thinning observed in frontal and cingulate regions might reflect an optimization process, potentially involving synaptic refinement, which aligns with previous studies proposing that reduced cortical thickness in certain areas can indicate more efficient neural circuitry (Holtmaat and Svoboda 2009; Magee and Grienberger 2020; Yoshihara et al. 2009; Zatorre et al. 2012). Mechanistically, these changes may involve glia- or synaptogenesis to support enhanced information processing and integration across sensory modalities (e.g., Zatorre et al. 2012). Our findings underscore the dynamic interplay between structural plasticity and functional connectivity following exposure-based audiovisual associations. They suggest that the brain adapts at both the network level and the structural level—strengthening integrative regions while refining control and modulatory circuits to support enhanced multisensory processing.

As mentioned above, the association phase of current design also included a behavioral measurement right after the association session on each day. Therefore, unisensory experience (visual motion) and training (direction discrimination) might also contribute to the observed changes in cortical thickness. For instance, it is conceivable that the decrease in cortical thickness in low- and/or mid-level visual areas might be driven by these additional measurement sessions and unisensory experience/training. Future research is needed to identify specific contributions of unisensory and multisensory experiences.

### Limitations and future directions

Our study aimed to capture the effects of exposure-based audiovisual associations on perceptual changes and neuroplasticity within a limited timeframe and without feedback, distinguishing our results from typical perceptual learning paradigms. Given this focus, our design did not include a control group, as prior literature robustly supports association-driven perceptual shifts and connectivity changes under similar conditions (e.g., Hidaka et al. 2011; Gonzalo et al. 2000). To overcome this limitation, we incorporated an untrained visual hemifield as a control region, enabling each subject to serve as their own control. Even though our behavioral findings suggest that the crossmodal associations themselves were dominant during the association phase, we also acknowledge that exposure to visual motion over the course of several days may contribute to the observed changes in functional connectivity and cortical thickness. Future studies incorporating a control group with unpaired stimuli or no association training will be informative in delineating general effects of visual experiences from crossmodal association-specific changes.

While our sample size is modest, it aligns with neuroimaging research in audiovisual perceptual learning (e.g., Gonzalo et al. 2000; Powers et al. 2012) and/or visual motion studies (Smith et al. 1998; Rina et al. 2022) where individual differences are minimized through a within-subjects design. We further applied FDR corrections to account for multiple comparisons and to reduce the risk of Type I error, ensuring our reported effects were robust. Future investigations with larger sample sizes will enable subgroup analyses to examine potential individual variability in response to crossmodal associations (e.g., correlation analyses) and provide a direct link between behavioral performance values and functional/structural alterations in specific brain regions. Specifically, with a larger sample size, it could be statistically feasible to calculate an index of audiovisual associations [e.g., (PSE_down, post_ − PSE_up, post_) − (PSE_down, pre_ − PSE_up, pre_)] and test whether participants who experienced a larger auditory influence/index in the post-training session also showed more pronounced changes in functional connectivity and cortical thickness. Given the modest sample size of current dataset, we have refrained from conducting such analysis. Nonetheless, the results of our primary statistical tests provide compelling evidence that passive (exposure-based) sensory experiences may lead to functional and structural plasticity in the adult brain.

It is also worthwhile to mention that previous research highlights the contributions of subcortical structures, such as the thalamus and superior colliculus, to audiovisual integration (for a recent review, see Gao et al. 2023). Notably, studies on humans have predominantly used spatial cueing paradigms (e.g., Fairhall and Macaluso 2009; van den Brink et al. 2014). Given that visual motion processing primarily relies on cortical areas, the paradigm used in this study may not optimally isolate subcortical contributions to audiovisual associations and sensory experiences. A more targeted approach, such as paradigms designed to elicit subcortical activation or studies involving specific patient populations (e.g., Laeng et al. 2021), would be beneficial for addressing this question.

## Conclusions

The current findings illustrate how passive exposure to audiovisual associations influence perceptual reports and lead to functional and structural changes in the adult brain. We found that audiovisual associations alter the behavioral reports on ambiguous visual motion, resulting in significant changes in coherence thresholds and highlighting the flexibility of mechanisms underlying dynamic vision. More importantly, resting-state recordings revealed enhancement of functional connectivity between our seed regions at early visual areas and higher-order cortical regions, alongside differential structural plasticity. The overlap between functional and structural measures indicates that enhanced connectivity aligns with structural expansion. These complementary findings highlight the brain's capacity to reorganize itself in response to multisensory input, strengthening integrative regions while refining control and modulatory circuits. Our results emphasize the dynamic nature of adult brain plasticity and the importance of crossmodal associations and correspondence in perception and adaptive behavior.

## Supplementary Information

Below is the link to the electronic supplementary material.

Supplementary file1 (DOCX 745 KB)

## Data Availability

The pre-processed fMRI data and behavioral data that support the findings of this study are available on request from the corresponding author.
