# Supplementary material for: Functional and structural plasticity induced by audiovisual associations and sensory experiences
Source: Brain Struct Funct. 2025 Jun 8;230(6):89. doi: 10.1007/s00429-025-02951-3 (PMC12146228; doi:10.1007/s00429-025-02951-3)
Supplement: Supplementary file 1 — Supplementary file1 (DOCX 745 KB) [file 429_2025_2951_MOESM1_ESM.docx]

Functional and Structural Plasticity Induced by Audiovisual Associations and Sensory Experiences

**Fazilet Zeynep Yildirim-Keles^1,2^, Pinar Demirayak^3,4^, Hulusi Kafaligonul^5,6^***

^1^Department of Psychology, Boğaziçi University, Istanbul, Türkiye

^2^Department of Psychology, University of Fribourg, Fribourg, Switzerland

^3^Civitan International Research Center, University of Alabama at Birmingham, Birmingham, AL 35233, USA

^4^Department of Neurobiology, University of Alabama at Birmingham, Birmingham, AL 35233, USA

^5^Neuroscience and Neurotechnology Center of Excellence (NÖROM), Department of Anatomy, Faculty of Medicine, Gazi University, Ankara, Türkiye

^6^Aysel Sabuncu Brain Research Center, Department of Neuroscience, Bilkent University, Ankara, Türkiye

***Corresponding author:** Hulusi Kafaligonul

**Contacts**

Fazilet Zeynep Yildirim-Keles: [fazilet.keles@bogazici.edu.tr](mailto:fazilet.keles@bogazici.edu.tr)

Pinar Demirayak: [pinarde@uab.edu](mailto:pinarde@uab.edu)

Hulusi Kafaligonul: [hulusi@bilkent.edu.tr](mailto:hulusi@bilkent.edu.tr) [hkgonul@gazi.edu.tr](mailto:hkgonul@gazi.edu.tr)

**SUPPLEMENTARY MATERIAL**


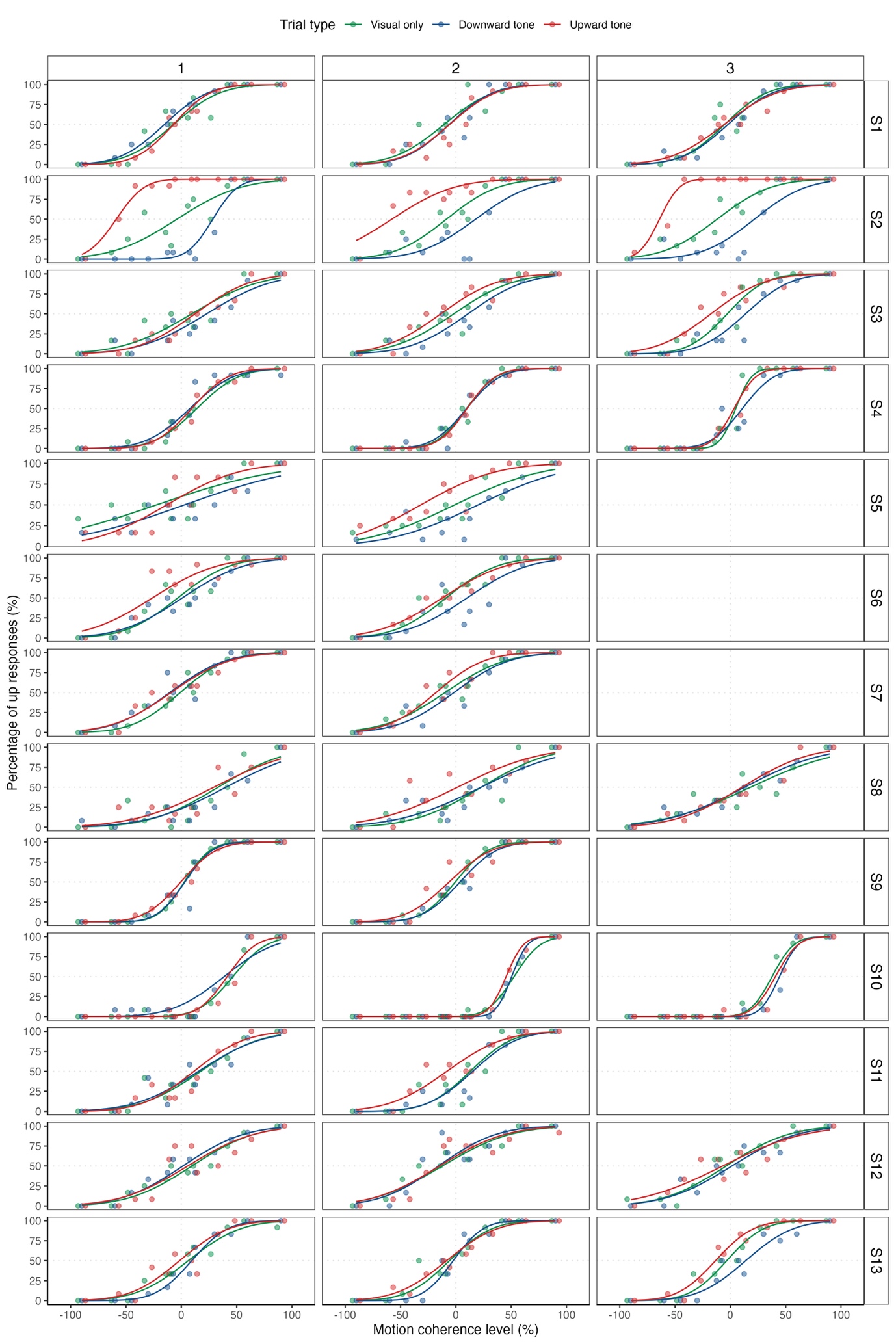


**Figure S1.** The behavioral measurements performed right after the association phase on each day. Each row and column display measurements from each subject on a specific training day, respectively. In terms of association induced changes in psychometric curves, while some subjects show an improvement over days (S3, S4, S5, S7, S9, S11, S13) others do not (S1, S2, S6, S8, S10, S12).


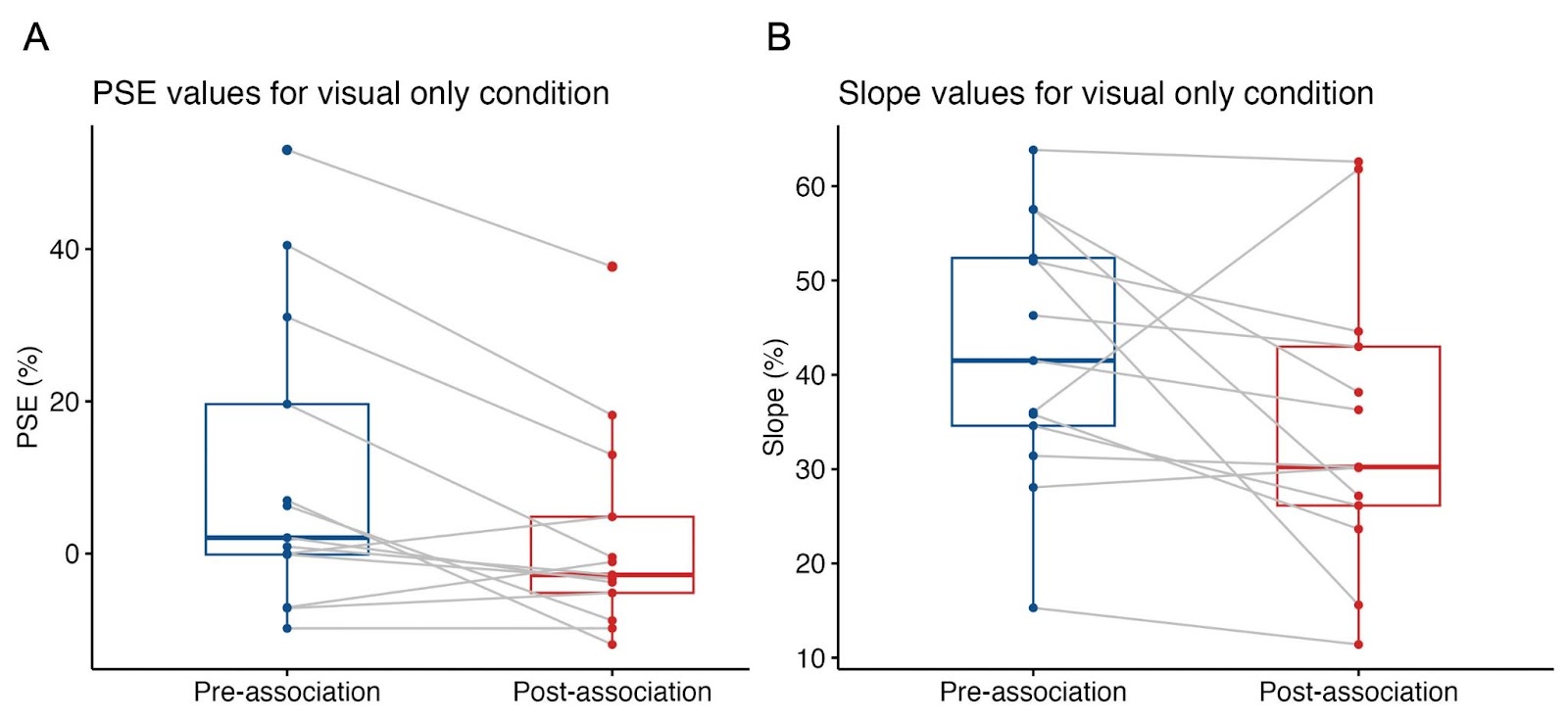


**Figure S2.** The changes in PSE and slope values for the visual only condition across pre- and post-association phases. **(A)** The PSE values significantly decreased (i.e., observers became less biased in reporting downward motion) in the post-association phase. **(B)** The slope values did not change between pre-and post-association phases (i.e., direction discrimination did not improve).
